# Folk nomenclature and traditional knowledge of breadfruit [*Artocarpus altilis* (Parkinson) Fosberg] diversity in four Anglophone Caribbean countries

**DOI:** 10.1186/s13002-022-00562-4

**Published:** 2022-11-10

**Authors:** Oral O. Daley, Laura B. Roberts-Nkrumah, Angela T. Alleyne, Michael C. Gloster

**Affiliations:** 1 Department of Food Production, Faculty of Food and Agriculture, The University of the West Indies, Saint Augustine, 330912 Trinidad and Tobago; 2 Department of Biological and Chemical Sciences, Faculty of Science and Technology, The University of the West Indies, Cave Hill, Barbados

**Keywords:** Folk taxonomy, Genetic resource management, Local knowledge, On-farm conservation

## Abstract

**Background:**

Since its introduction to the Anglophone Caribbean in 1793, breadfruit has had a diverse history in the region, and there is a considerable repository of traditional knowledge about the crop, that is undocumented. Consequently, it remains underutilized as a food source, despite recognition of its potential to contribute to food and nutrition security. Understanding the folk taxonomy and traditional knowledge associated with its diversity and uses is a prerequisite to develop programs for its commercial production and utilization.

**Method:**

This study was conducted among 170 respondents who were selected across four Anglophone Caribbean countries and provided information on the ethnobotany and traditional knowledge associated with breadfruit biodiversity, including systems of naming, identification and classification of breadfruit cultivars or types.

**Results:**

Breadfruit has socio-cultural and economic value and is produced for both home use and sale by most respondents (68%). The genetic diversity of breadfruit managed by the respondents is also important, as a total of 51 vernacular names were identified, with nine of those names recorded for the first time in this study. Breadfruit types were identified by morphological and agronomical characteristics, with other important traits relating to use and cooking quality. Classification of breadfruit cultivars or types was based on eating-quality, most suitable methods of preparation and ease of cooking.

**Conclusion:**

The ethnobotanical and traditional knowledge obtained from this study may be useful in assessing the genetic diversity of breadfruit and guiding future community-based conservation and classification studies of this important crop resource in the Caribbean. This is crucial to support the commercialization of breadfruit to improve its contribution to food and nutrition security.

## Introduction

Breadfruit [*Artocarpus altilis* Parkinson (Fosberg) was domesticated in Oceania but is now widely distributed throughout the tropics [1]. In 1793, the British sea captain William Bligh successfully transported 682 breadfruit plants along with other plants of economic importance from Tahiti and Timor to the British Caribbean islands [2]. French voyagers also collected one seedless breadfruit in Tonga, which was distributed to the French Caribbean islands in the 1790s [3]. The introduction of breadfruit was envisaged to help reduce food shortages that severely affected the region, and breadfruit was considered the ideal crop based on earlier reports by other European explorers in the Pacific, who saw it as an easy crop to produce and a reliable source of food in the Pacific islands [4, 5]. The breadfruit plants introduced by Bligh were delivered directly to the islands of St. Vincent and Jamaica from which planting materials were subsequently distributed to other territories of the British Caribbean [6]. However, planting was mainly done on marginal lands to avoid competition with sugarcane (*Saccharum officinarum*), which was the main economic crop produced throughout the region during the 18th century [5].

Breadfruit was not immediately favored for human consumption by the local population and periodically became a major source of food only after Emancipation in 1834 [6]. Consequently, the crop has played important roles in food and nutrition security and livelihood for many householders, especially those in rural communities [6–8]. However, its commercial potential has not been fully explored, and it is not generally regarded as a crop of significant economic importance [9]. It is worth noting that it is not included in the agricultural statistics for several Anglophone Caribbean countries where it is largely underutilized despite the high food import bill in these countries [9].

Although several breadfruit varieties introduced to the Anglophone Caribbean in the 1990s have been clearly identified and characterized, the diversity of the original germplasm distributed throughout this sub-region remains unknown [9]. The current geographical distribution of some breadfruit varieties can still be traced to areas of the Pacific, where they were originally collected by European explorers including Captain Bligh. However, Bligh did not identify the varieties he introduced to the Caribbean in 1793, but reported that he had five seedless types from Tahiti and two from Timor (one seedless and one seeded type) [2]. On an earlier ill-fated attempt in 1789 to introduce the breadfruit to the Caribbean, Bligh recorded the names of eight cultivars that he had collected: ‘Appeere’, ‘Awanna’, ‘Eroroo’, ‘Mire’, ‘Oree’, ‘Patteah’ ‘Powerro,’ and ‘Rowdeeah’ [10]. Both sets of plants were collected from the same source, and it is likely that materials introduced in 1793 were from among the same eight cultivars recorded in Bligh’s first voyage [2, 4].

In the St. Vincent Botanical Garden, where some of the plants introduced by Bligh were planted, the garden curator described six seedless breadfruit varieties, which were distinct in seasonality, fruit size and shape [11]. Leakey [3] reported five seedless varieties of breadfruit found in St. Vincent followed later by Andrews and Mason Jr. [12], who described seven breadfruit types, while Roberts-Nkrumah [13] reported 25 cultivar names in a survey of the island. Breadfruit was distributed from the botanical gardens in St Vincent to most of the other British territories during the 18th and 19th century. Tobago was an early recipient, with the planter, John Robley being awarded a gold medal in 1802 by The Royal Society of Arts for successfully establishing trees there [4].

In Jamaica, where almost half of the original plants brought to the region were delivered Weir, Tai [14] recorded four cultivars, Webster [15] also described four cultivars but with some differences in cultivar names, and Roberts-Nkrumah [16] reported eight cultivar names. Andrews and Mason Jr. [12] also reported four named cultivar names in Grenada and three each in St Lucia and Dominica.

Preservation and transmission of traditional knowledge of breadfruit biodiversity, production, utilization and conservation are essential for the promotion of breadfruit for food and nutrition security in the Anglophone Caribbean [8]. Limited documentation of this traditional or localized knowledge, which often relies on oral transmission from one generation to the next, may likely contribute to the underutilization of the crop in the region. In the Pacific, traditional knowledge was deemed the most valuable tool for cultivar identification of breadfruit, and the disappearance of many cultivars was related to the inter-generational loss of this knowledge [17]. Furthermore, by understanding traditional knowledge associated with breadfruit, researchers in the Republic of Marshall Island (RMI) discovered that two local cultivars were neglected and were threatened by extinction because they bore smaller-sized fruits and were not as prolific as other cultivars [18]. As food supply and consumption become more globalized, knowledge accumulated over millennia for underutilized crops such as breadfruit could disappear in a few generations, even in very remote areas, if it is not documented [19, 20]. Details of the traditional uses of breadfruit are available for the Pacific region [21]. Navarro, Malres [22] indicated that due to less oral transmission than in the past, significant loss of traditional knowledge of breadfruit uses, was associated with significant loss of genetic diversity. Detailed descriptions of breadfruit uses and information on its cultural significance and relevant varieties have not been found for the Anglophone Caribbean. Roberts-Nkrumah and Legall [8] described some of the uses of breadfruit in Trinidad and Tobago, and consumer preferences between two breadfruit cultivars based on sensory characteristics and preparation methods have also been described [23]. Documentation of traditional knowledge of breadfruit is important to increased utilization, and consequently, production and conservation of the existing biodiversity of this crop in the Anglophone Caribbean. Therefore, the objectives of this study were to present information on folk nomenclature and traditional knowledge associated with breadfruit diversity and to investigate systems of naming, identifying, and classifying breadfruit cultivars or types in the Anglophone Caribbean.

## Methods

### Area of study

**Fig. 1:**
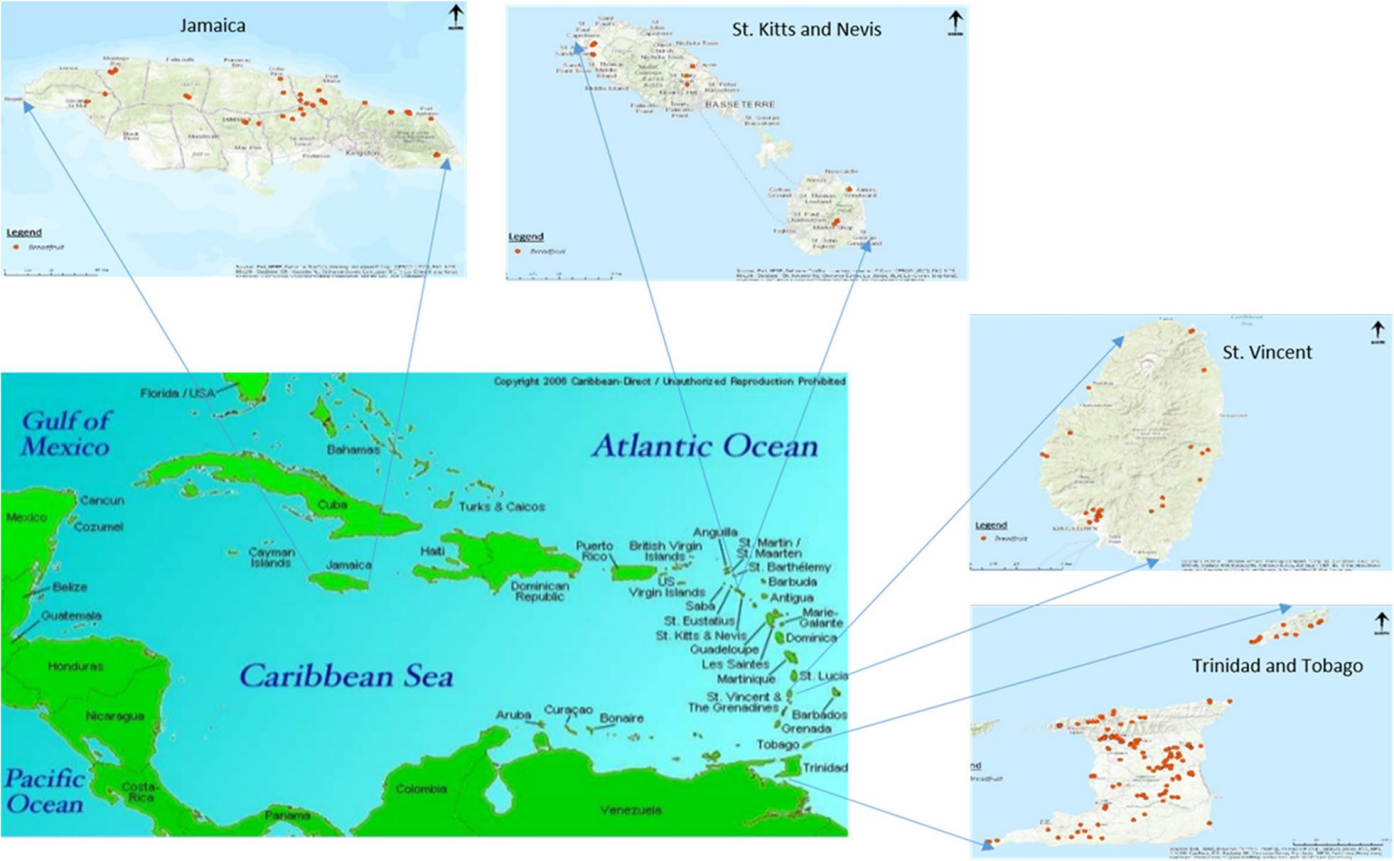
Map of the Caribbean highlighting countries surveyed

The Anglophone Caribbean consists mostly of islands in the Greater and Lesser Antilles in a chain located southeast of North America and includes mainland countries in eastern Central America and north-western South America, all wholly or partially washed by the Caribbean Sea. A survey was conducted in four countries of the Anglophone Caribbean namely, Jamaica in the Greater Antilles and St. Vincent and the Grenadines, St. Kitts and Nevis, and Trinidad and Tobago in the Lesser Antilles (Fig. 1). In St. Vincent and the Grenadines, the survey was conducted only on the main island of St. Vincent., while data from the two islands of St. Kitts and Nevis were combined because of the small sample size from both islands, and Trinidad and Tobago were treated as two separate islands. These countries were selected based on accessibility to key informants, historical associations with breadfruit importation to the Caribbean and documentation of breadfruit production activities. They also represent different sub-regions within the wider Caribbean.

### Data collection

Between 2012 and 2015, ethnobotanical information was collected among 170 respondents in the four countries (Fig. 1). For each country, an initial list of potential respondents with their locations and telephone numbers was prepared consisting of breadfruit tree owners who were either farmers or homeowners or both, and other persons knowledgeable about breadfruit who were already known to at least one of the researchers from previous surveys [7, 8, 13] as in St. Vincent, Jamaica, and Trinidad. A farm, in the context of these countries, is an area of land cultivated by an individual or a family. Persons knowledgeable about breadfruit were mainly extension officers of the Ministry or Department of Agriculture who lived or worked in the parishes or counties of the country, or elderly consumers. These lists were updated by contacting the potential respondents directly by telephone to confirm their availability and willingness to participate in an interview and to request their assistance in identifying other potential respondents. Therefore, those who identified other potential respondents were also informants. Other informants were extension officers who had not been involved in previous surveys but knew breadfruit tree owners who were not already on the list. The surveys began with persons who had agreed to participate in the interviews at a mutually convenient date and time. The list continued to be updated using the snowball sampling technique as other informants were encountered, for example, shopkeepers or residents in a district who did not know about breadfruit varieties themselves but suggested the names of persons knowledgeable about breadfruit or those who owned trees. Where possible, the extension officer or other informant who was known to the respondent, introduced the researcher to the respondent. The survey also incorporated the experiences of all researchers who grew up in the region and were able to use their experience to identify tree owners and persons knowledgeable about breadfruit. Before all interviews, the potential respondents were advised about the affiliation of the researchers, the nature of the information that was being requested, the purpose for which it was being collected and assurance that their names and contact information would not be shared or published. Their willingness to participate was confirmed again. No inducement or payment was made for respondent participation.

Interviews were conducted using a semi-structured questionnaire, administered by the researchers, and consisted of the following open-ended questions:

1. What are the names of breadfruit cultivars or types that you know?
2. How do you identify and describe the breadfruit cultivars or types that you know?
3. How do you use the breadfruit cultivars or types that you know?
4. Do you know of other uses for breadfruit?

The responses were recorded as summarized written notes. Where trees were accessible, photographs were taken, and leaf, flower and fruit samples were collected and placed in labelled bags for measurement within five hours. Interviews in a district, county or parish were discontinued when no other respondents were available or could add no new information.

### Data analysis

Descriptive and inferential statistical data analyses were carried out using IBM SPSS Version 21 [24]. Descriptive statistical methods included frequencies, percentages, and means. Inferential statistical methods included Chi-square test of association. The information on varietal names and uses were summarized in tables for each country or island.

## Results

### Demographics of respondents

Table 1 provides a summary of the number and demographics of respondents in the survey. One hundred and seventy respondents were interviewed throughout the study. Respondents from Jamaica and Trinidad comprised half of the respondents (26% and 24%, respectively). Most were male (58%). Most respondents were from rural areas (54%) compared with those from semi-urban (31%) and urban areas (15%).

**Table 1:** Demographics of respondents in the survey

Island	Respondents					
	Gender			Location		Island total (%)
	Male (%)	Female (%)	Urban (%)	Semi-urban (%)	Rural (%)	
Jamaica	32 (71)	13 (29)	5 (11)	13 (29)	27 (60)	45 (26)
St. Kitts	6 (46)	7 (54)	1 (8)	3 (23)	9 (69)	13 (8)
St. Vincent	19 (58)	14 (42)	5 (15)	8 (24)	20 (61)	33 (19)
Tobago	18 (46)	21 (54)	5 (13)	15 (39)	19 (48)	39 (23)
Trinidad	23 (58)	17 (42)	9 (23)	14 (34)	17 (43)	40 (240
Respondents total	**99 (58)**	**71 (42)**	**25 (15)**	**53 (31)**	**92 (54)**	**170 (100)**

### Breadfruit production systems

Based on respondents’ responses in this survey, breadfruit was produced in four types of production systems. These were border plantings, home gardens, mixed cropping and pure stands or monoculture orchard. Border plantings, characterized as single or scattered trees planted along the boundaries of farms and home gardens (35%), and trees in home gardens (34%) represented the two most common breadfruit production systems. This was followed by mixed cropping with other perennial or annual crops (29%) (Table 2). Pure stand breadfruit orchards, though rare (2%), were observed in two locations in Jamaica and one location in Trinidad. There was no significant association (*χ*^*2*^ = 15.862, df = 12, *p* = 0.198) in the distribution of production systems among countries/islands.

**Table 2:** Breadfruit production systems based on respondents in the Anglophone Caribbean

Country/Island	Production system [No. of respondents (% within island)]				Total (%)
	Border planting (%)	Home gardens (%)	Mixed cropping (%)	Pure stand (%)	
Jamaica	16 (36)	18 (40)	9 (20)	2 (4)	**45 (26)**
St. Kitts	8 (61)	4 (31)	1 (8)	0 (0)	**13 (8)**
St. Vincent	12 (36)	7 (21)	14 (42)	0 (0)	**33 (19)**
Tobago	11 (28)	17 (44)	11 (28)	0 (0)	**39 (23)**
Trinidad	13 (32.5)	12 (30)	14 (35)	1 (2.5)	**40 (24)**
Total	**60 (35)**	**58 (34)**	**49 (29)**	**3 (2)**	**170 (100)**

**Fig. 2:**
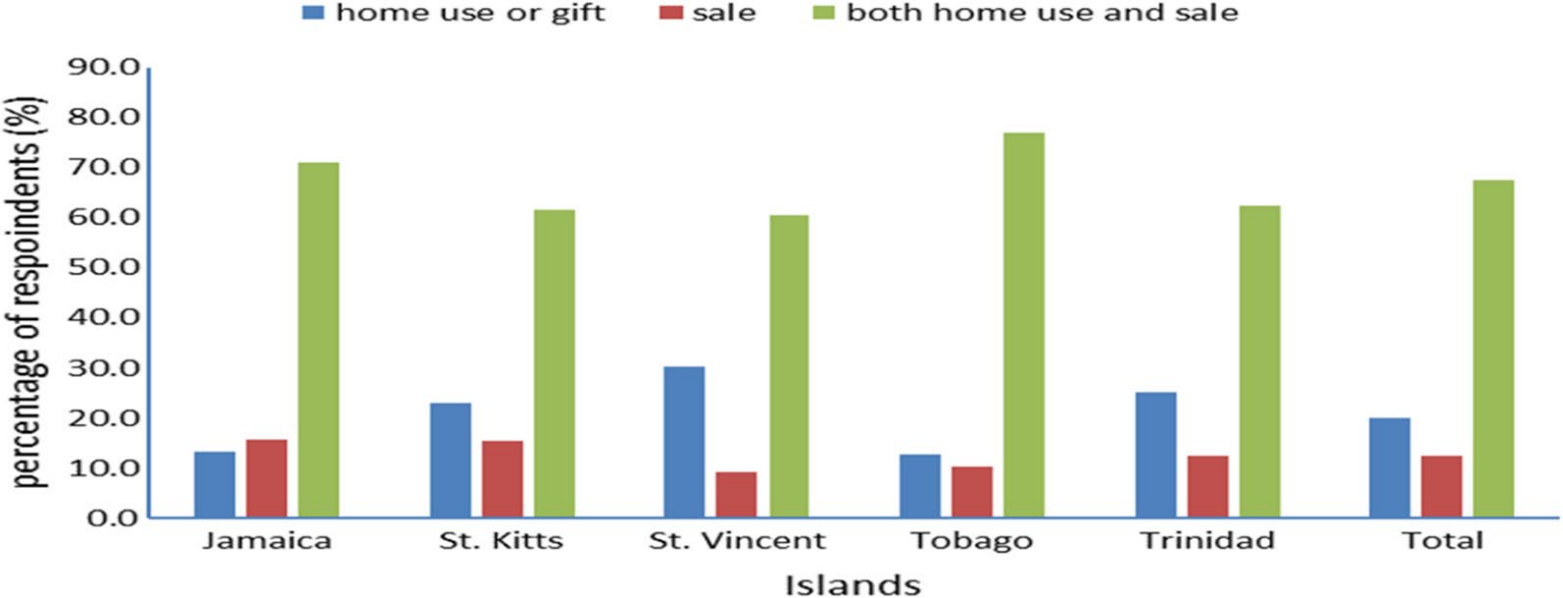
Respondents’ reasons for cultivating breadfruit in the Anglophone Caribbean

Breadfruit cultivation for food, sharing with relatives, friends, and neighbors or for household sales was important in the region. Respondents cultivated breadfruit in their home gardens or farms for home use or sharing with relatives, friends, and neighbors only (20%), for sale only (12.4%) or both home use or sharing with relatives, friends, and neighbors and sale (67.6%) (Fig. 2). Home use and individual sales were the most frequent reasons for cultivation and did not differ significantly among the islands (*χ*^*2*^ = 6.26, df = 8, *p* = 0.618).

### Breadfruit diversity and descriptive vernacular names used in the Anglophone Caribbean

In the present study, 38 vernacular names of breadfruit were reported by respondents among the countries surveyed. The largest number of cultivar names were recorded in St. Vincent (23), followed by Jamaica (15), Tobago (4), Trinidad (2) and St. Kitts and Nevis (2). Four of these cultivar names were shared among islands, while several names were used on only one island (Table 3). Twenty-one of the 23 cultivar names identified in St. Vincent were recorded only on that island. Similarly, 13 of the 15 vernacular names recorded for Jamaica were not shared with any other island in the survey. One unique vernacular name, ‘Chouf chouf’, was recorded for Tobago. All cultivar names used in Trinidad and St. Kitts were either shared with other islands or between those two islands.

**Table 3:** Breadfruit vernacular names and descriptions in the Caribbean as reported in the literature, and observed in in situ collections and recorded in the present study

No	Vernacular names	Country/Island	Descriptions based on respondents	Descriptions based on literature search	References
1	Banbran	Jamaica	ND	Fruits are round and small	[3, 16]
2	Banjam	Jamaica	Fruits are large with white to pale yellow flesh and very rough skin, bear throughout the year and easy to cook	ND	[16]
3	Black Breadfruit	St. Vincent and the Grenadines	Fruits are large, round with smooth skin	Fruit are oval with relatively rough and light green skin	[13]
4	Bois Pain	St. Lucia	ND	Medium-sized fruit with yellow flesh	(12)
5	Brambram*	Jamaica	Large fruits with smooth textured pulp, very rough skin and easy to roast	ND	ND
6	Butter Breadfruit	St. Vincent and the Grenadines	Small to large round fruit. Pulp color is very yellow and excellent texture	Fruits are subglobose, have relatively rough green skin	[13]
7	Butterheart	St. Vincent and the Grenadines and Tobago	Small to large round fruit. Pulp color is very yellow and excellent texture	Fruits are subglobose, have relatively rough green skin	[13]
8	Captain Bligh	St. Vincent and the Grenadines	Medium-sized fruit with yellow pulp and excellent for cooking. Leaves have very deep sinuses	Subglosoe fruit with fairly rough and light green skin. Leaves have thin lobes	[3, 12, 13]
9	Cassava	Jamaica	Medium sized oval fruit with a yellow pulp. Easy to cook. Leaves have very deep sinuses	Small to medium size fruit with pale yellow pulp with good flavor and long shelf life. Leaves have deep sinuses, and sometimes there is sub-lobing	[15, 16, 31]
10	Cocobread	St. Vincent and the Grenadines	Very large fruit with smooth skin, easy to cook and has an excellent mouth-feel	Large oval fruit, with very smooth skin and yellow flesh. Wrinkled leaves	[3, 12, 13]
11	Common	St. Vincent and the Grenadines	Smooth skin, yellow flesh, easy to cook. Very popular	Smooth to rough skin, yellow fleshed fruit	[3, 13, 16]
12	Chouf chouf*	Tobago	Round, medium-sized fruit with very rough skin and creamish to white pulp that is easy to cook	ND	ND
13	Couscous*	Jamaica	Medium to large round or oval fruit with very rough skin. Creamish to a pale-yellow pulp. Easy to cook. Leaves are wrinkled	ND	ND
14	Creole	St. Vincent and the Grenadines	Smooth skin, yellow flesh, easy to cook. Very popular	Round smooth, light green skin	[12, 13]
15	Dessert	St. Vincent and the Grenadines	Large fruit with thick yellow pulp and very easy to cook	Oval fruit with fairly smooth yellow green skin	[13]
16	England	St. Vincent and the Grenadines	Medium-sized fruit with yellow pulp and excellent for cooking. Leaves have very deep sinuses	Subglosoe fruit with fairly rough and light green skin. Leaves have thin lobes	[3, 12, 13]
17	Finey*	Jamaica	Small to medium fruit, round or oval fruit with yellow to bright yellow pulp. Easy to roast and of very good eating-quality	ND	ND
18	Floater	St. Vincent and the Grenadines	Large fruit with smooth skin and yellow pulp	Large round fruit with smooth skin and yellowish cream pulp	[13]
19	Green Skin	Grenada	ND	Medium-sized fruit, yellow flesh	[12]
20	Hard Nature*	St. Vincent and the Grenadines	Medium sized rough-skinned fruit with white to a creamish pulp. Hard to cook	ND	ND
21	Hogpen	St. Vincent and the Grenadines	Large round fruit, smooth skin, thick yellow pulp. Easy to cook	Medium sized round fruit with smooth, light green skin, yellowish cream pulp	[13]
22	Hope Marble	St. Vincent and the Grenadines	Small round fruit with smooth skin, and yellow to very yellow pulp. Very easy to cook	Small, subglobose fruit with smooth green skin and light-yellow pulp	[13, 16, 31]
23	Kashee Bread	St. Vincent and the Grenadines	Medium to large oval fruit, with very, very rough skin and yellow flesh. Leaves are lopsided and drooping	Medium sized, spiny fruit; yellow flesh. Wrinkled or puckered and drooping leaf	[12, 13]
24	Kele kele	French colonies in the Caribbean	ND	Seedless	[3]
25	Koshi	St. Vincent and the Grenadines	ND	Large, round, and rough skinned. Yellow flesh with excellent eating-quality	[3]
26	Lawyer Caine	St. Vincent and the Grenadines	Medium sized, oval fruit with slightly yellow pulp. Fairly easy to cook	Medium sized, round fruit with smooth, light yellow green skin and yellowish cream pulp	[13]
27	Liberal	St. Vincent and the Grenadines	Round fruit with creamish pulp. Easy to cook. Bears off-season	Oval fruit with fairly rough, light green skin and pale cream pulp	[13]
28	Lulu	St. Vincent and the Grenadines	Very large fruit, with rough skin. Easy to cook and roast	Large, oval with rough to spiny green skin and whit to cream pulp	[13]
29	Macca	Jamaica	Large fruits with smooth, soft textured, pale yellow-to-yellow pulp, very rough skin and easy to roast. Has good eating-quality	Fruits are medium to large, round or oval and skin has very pronounced raised polygons resulting in a rough surface. Pulp color if pale yellow to bright yellow with good eating-quality	[15, 16, 32]
30	Man Bread	Jamaica	Large oval fruit with smooth to slightly rough skin, yellow to very yellow pulp and easy to cook	ND	ND
31	Mary Grace*	St. Vincent and the Grenadines	Round or oval fruit, with smooth skin, creamish pulp with firm texture and good for roasting	ND	ND
32	Massa	St. Vincent and the Grenadines	Large, round, smooth skin and white pulp	Medium-sized fruit white flesh; smooth to slightly rough	[12, 13]
33	Monkey Breadfruit*	Jamaica	Round, medium-sized fruit with very rough skin and creamish to yellow pulp that is easy to cook	ND	ND
34	Old wind	St. Vincent and the Grenadines	ND	Medium sized round fruit with smooth, light yellow-green skin and yellowish cream pulp	[13]
35	Ordinary	St. Lucia	ND	Medium-sized fruit, yellow flesh	[12]
36	Pika	Grenada	ND	Medium sized, spiny fruit with yellow flesh. Wrinkled or puckered and drooping leaf	[12, 16]
37	Prickly Walled	Jamaica	ND	Fruit skin has elongated soft spines, and the cultivar is not very common	[14]
38	Ready Roast	St. Vincent and the Grenadines	Medium to large fruit with thick yellow pulp and very easy to cook	Oval fruit with fairly smooth yellow-green skin	[13]
39	Red Bread*	St. Vincent and the Grenadines	Large, oblong fruit with slightly rough skin and thick very yellow pulp	ND	ND
40	Sally Young	St. Vincent and the Grenadines	Very large, round fruit with smooth to rough skin and creamish pulp. Easy to cook	Fruit are oval with slightly rough, light green skin and pale color pulp	[13]
41	Soursop	St. Vincent and the Grenadines	Medium-sized fruit with very rough skin. White pulp and difficult to cook. Leaves are narrow and dull	Fruits are oval, with slightly rough, light green skin and pale cream pulp	[13]
42	Smooth Skin*	St. Vincent and the Grenadines	Medium to large fruit with thick yellow pulp and very easy to cook	ND	ND
43	St. Kitts	Jamaica	Round, small to medium fruit with light yellow to yellow pulp, easy to cook. Leaves range from having one or two short lobes on the upper one-third to having no lobes	Fruits are oval or round and small to medium with pale yellow pulp. Leaves are entire with dentate margin	[14, 15]
44	Timor	Jamaica	Round, small to medium fruit with light yellow to yellow pulp, easy to cook. Leaves range from having one or two short lobes on the upper one-third to having no lobes	Fruits are oval or round and small to medium with pale yellow pulp. Leaves are entire with dentate margin	[31]
				Fruits are small and very inferior. Leaves have very deep sinuses almost reaching the midrib	[3, 29]
45	White	St. Vincent and the Grenadines, Grenada and St. Lucia	Large, round, smooth skin, and white pulp	Medium-sized fruit white flesh; smooth to slightly rough	[12, 13]
46	White Heart	Jamaica	Large, round, smooth skin, and white pulp	Small to very large fruit with white pulp, difficult to cook and poor flavor	[14, 15]
47	Yam Paine Blanc	Dominica	ND	Light green skin and cream flesh	[12]
48	Yam Paine Common	Dominica	ND	Medium-sized fruit, yellow flesh	[12]
49	Yam Paine Juane	Dominica	ND	Medium-sized fruit, yellow flesh	[12]
50	Yellow	Trinidad and Tobago and St. Kitts and Nevis	Medium to large fruit, round or oblong fruit with pale to bright yellow pulp. Easy to roast and have good eating-quality	Medium to large fruit, round or oblong fruit with pale to bright yellow pulp. Easy to roast and have good eating-quality	[31]
51	Yellow Heart	Jamaica	Medium to large fruit, round or oblong fruit with pale to bright yellow pulp. Easy to roast and have good eating-quality	Medium to large fruit, round or oblong fruit with pale to bright yellow pulp. Easy to roast and have good eating-quality	[14, 15]

*Vernacular names first reported in this study, *ND* No data

When vernacular names recorded in this survey were combined with those reported in earlier studies, a total of 51 different names were known throughout the Caribbean (Table 3). Nine unique vernacular names were recorded for the first time in this survey. These included ‘Brambram,’ ‘Couscous,’ ‘Finey’ and ‘Monkey breadfruit’ from Jamaica, ‘Mary Grace,’ Hard Nature,’ ‘Red Bread,’ and ‘Smooth Skin’ from St. Vincent and ‘Chouf chouf’ from Tobago (Table 3).

### Folk nomenclature and identification of breadfruit types in the Anglophone Caribbean

Respondents who were knowledgeable of breadfruit diversity used different approaches to identify, name, and then classify breadfruit types or cultivars. For identification, respondents in this survey used 16 descriptors related to plant morphological characteristics (skin texture, fruit size, fruit shape, skin color, pulp color, leaf shape) and agronomic characteristics (time of bearing, time to maturity) (Table 4). Respondents perceived a range of breadfruit types, each having distinct features, and they used a combination of descriptors to identify breadfruit types or cultivars. The most frequently mentioned descriptor used for the identification of breadfruit was pulp color (77%), followed by skin texture (58%), fruit size (30%) and leaf shape (25%) (Table 4).

**Table 4:** Descriptors used by respondents for the identification of breadfruit  cultivars in the Caribbean

Identification and characterization criteria	Respondents* (%)	Criterion category	Cultivar names
*Plant morphology*			
Pulp color	77	White	White, White Heart, Captain Bligh
		Cream	Macca
		Light yellow	Timor, St. Kitts
		Yellow	Yellow Heart, Butterheart
Skin texture	58	Smooth	Black Breadfruit, Smooth Skin, White, White Heart, Yellow Heart
		Sandpapery	Red Bread
		Rough/spiny	Macca, Choufchouf, Waterloo, Monkey Breadfruit
Fruit size	30	Large	Black Breadfruit, Sally Young, Waterloo
		Medium	Lawyer Caine, Soursop
		Small	Hope Marble
Leaf characteristics	25	Deeply lobed	Cassava
		Moderately lobed	White Heart, Yellow Heart
		Slightly lobed on the upper one-third of the leaf to entire	Timor, St. Kitts
Fruit shape	16	Round	Lawyer Caine, Dessert
		Oblong	Red Bread
		Irregular	Choufchouf
Skin color	10	Brown	Yellow Heart
		Green	Green Skin, Soursop
		Yellow green	Kashee
Core size	5	Large	Brambram, Banjam
		Small	Hogpen, Dessert
*Agronomic characteristics*			
Time of bearing	15	June to September	Yellow Heart
		December to February	
		Year round	Liberal, St. Kitts
Time to fruit maturity	4	Fast	Yellow Heart
		Slow	Cassava
*Other*			
Frequency of occurrence	5	Common	Common, Creole, Ordinary, Yellow
		Rare	Couscous, Choufchouf, Brambram

*Percentage of respondents using identification criteria for distinguishing breadfruit cultivars

The pulp color states identified were white, cream, light yellow and yellow. The yellow pulp color of the cultivar ‘Yellow’/’Yellow Heart’ is often used as a benchmark for comparison with other cultivars. For example, a respondent referring to the pulp color of ‘Timor’ in Jamaica suggested that the pulp was not as yellow as ‘Yellow Heart.’ This was interpreted to mean that ‘Timor’ had a light-yellow pulp. Skin texture is also an important feature in cultivar identification and naming. Cultivars such as ‘Macca’ in Jamaica, ‘Kashee Bread in ‘St. Vincent’ and ‘Chouf chouf’ in Tobago were all identified first by their very rough skin. The names given to these cultivars are also based on their rough to spiky skin.

**Fig. 3:**
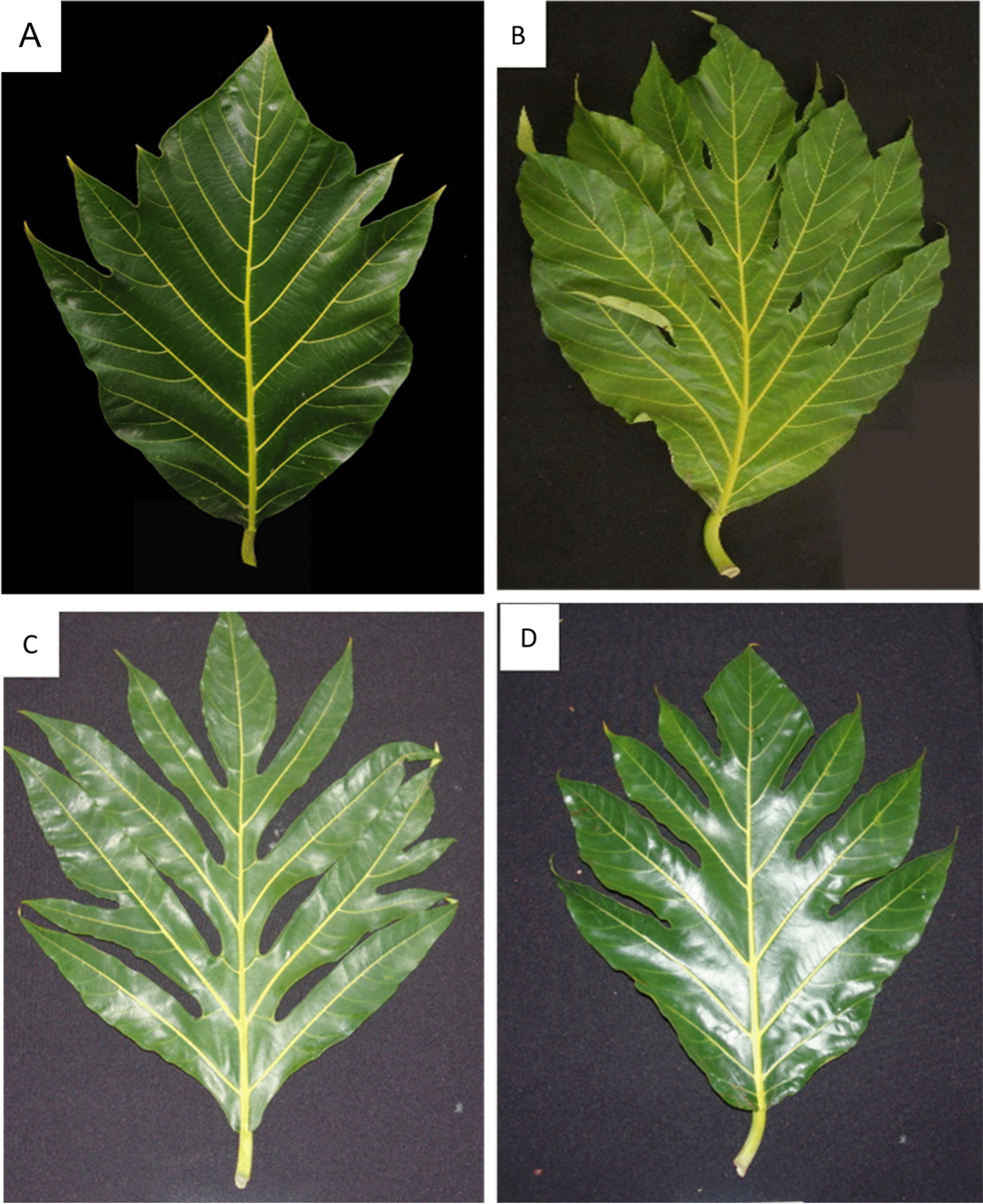
Leaf morphological variations observed among breadfruit cultivars in the Anglophone Caribbean: (**A**) Timor/ St. Kitts; (**B**) Kashee Bread; (**C**) Cassava, and (**D**) Yellow.

Leaf shape was also used to describe cultivars, and in some cases, it was the first descriptor used for those with distinct leaves (Fig. 3). For example, cultivars ‘Cassava’ in Jamaica and ‘Captain Bligh’ in St. Vincent were readily identified by their leaves, which had very deep sinuses (Fig. 3). In Jamaica, the names ‘Timor’ and ‘St. Kitts’ refer to the same cultivar, but both names were never used in the same location. However, when asked to give key identifying characteristics, respondents who used those names always gave the description for a cultivar with an unusual entire or dentate margin on the upper one-third of the leaf.

### Traditional knowledge of breadfruit names and their classification in the Anglophone Caribbean

Many of the vernacular names encountered in this survey were often descriptive and reflected variation in fruit morphology, cooking and eating-quality and association with people, places, and things (Table 5). Some of these names were passed down from generation to generation and used by respondents without an understanding of their meaning. However, there were other names for which respondents perceived meaning. Vernacular names such as ‘Timor,’ ‘St. Kitts’ and ‘England’ were all based on the names of places (Table 5). Some vernacular names were based on people associated with the specific cultivar or type. For example, the vernacular name ‘Captain Bligh’ was based on the name of the sea captain who introduced breadfruit to the Anglophone Caribbean. Vernacular names such as ‘Yellow Heart,’ ‘Yellow,’ ‘Creole,’ and ‘Common’ appear to describe the same cultivar. ‘Yellow Heart’ was recorded in Jamaica, ‘Yellow’ was recorded in Trinidad, Tobago, and St. Kitts, while ‘Creole’ and ‘Common’ were both recorded for St. Vincent. Similarly, the vernacular names ‘Cassava’ and ‘Captain Bligh’, appeared to describe the same cultivar based on morphological features. Even within the same island or country, different vernacular names were used to describe cultivars that appeared to be identical. In Jamaica, the vernacular names ‘Timor’ and St. ‘Kitts’ were used for the same cultivar, which is usually distinguished from other cultivars as having an unusual leaf shape.

**Table 5:** Breadfruit vernacular names and implications for their meaning in the Anglophone Caribbean

Naming of cultivars	Vernacular names and implications for their meaning
Based on names of places	Timor: This cultivar is believed to have be the Timoran cultivar introduced by Captain William Bligh) St. Kitts: In some parts of Jamaica, this cultivar is believed to be introduced from the island of St. Kitts England: Cultivar named after the country England which introduced Breadfruit to the Caribbean
Based on names of people	Sally Young (name of a local citizen of St. Vincent for which the variety became associated with) Mary Grace (name of a local citizen of St. Vincent for which the variety became associated with) Captain William Bligh: Cultivar named after the sea captain that introduced breadfruit to the Caribbean
Based on names of names of other plants	Cassava (Implying the cultivar has leaf with very deep lobes similar to the plant Cassava) Soursop (implying the cultivar has rough skin similar to Soursop)
Based on frequency of occurrence	Common, Creole and Ordinary (These names suggest that the cultivar is common seen and used or is much acquainted
Based on locally used words	Macca, Kashee (These words mean thorns in Jamaica and St. Vincent respectively. As cultivar names, they refer to the thorny appearance and feel of the skin of these cultivars
Based on typical use	Dessert (the fruit is considered to have good quality to be used for dessert) Hog Pen (this cultivar is used to feed pigs because of poor quality)
Based on names of other food items	Butter, Butter Heart (refers to the soft, smooth texture of the fruit pulp. It also relates to the similarity in color between the pulp color and yellowness of butter
Based on ease of cooking	Ready Roast (implies easy to roast) Hard Nature (means a hardy variety that is hard to cook) Hard to Roast (implies difficult to roast)
Based on pulp color	Yellow, Yellow Heart, Butter Heart, White, White Heart (implies cultivar with yellow or white pulp color)

Based on respondents in this survey, three criteria were used to classify breadfruit cultivars in the surveyed countries. These were eating-quality, the most suitable method of preparation and ease of cooking (Table 6). Moreover, three types of breadfruit were distinguished based on eating-quality, namely, ‘excellent’, ‘good’ and ‘poor’ (Table 6). Cultivars or types with excellent eating-quality usually had pulp that are yellow, soft, smooth texture when cooked and are often described as having a great mouth-feel. Cultivars with good eating-quality generally have cream to light yellow pulp and are described as having good mouth-feel and flavor. Poor eating-quality among breadfruit means firm pulp that is dry and has poor flavor. These cultivars generally have white to cream-colored pulp. Words such as ‘stringy,’ ‘barky’ and ‘strany’ were often used to describe the mouth-feel of those cultivars considered to have poor eating quality.

**Table 6:** Respondents’ classification of breadfruit cultivars in the Anglophone Caribbean

Identification and characterization criteria	Criterion category	Characteristics of each category	Examples of cultivars given by respondents
Eating—quality	Excellent	Gives a pleasant/smooth mouth-feel and flavor and may even be consumed without any protein food	Cocobread, Kashee Bread, Dessert, Yellow Heart
	Good	Good texture mouthfeel and flavor	Couscous, Macca
	Poor	Has a hard texture and poor mouthfeel. Often described as stringy	White, Soursop, Hard Nature, Hard to Roast
Most suitable method of preparation	Roasting	Roasts easily and has good flavor and texture	Yellow Heart, Easy roast, Butter, Dessert
	Boiling	More suited to boiling than roasting	White Heart, Couscous, Banjam
Ease of cooking (roasting or boiling)	Easy	Cooks very easily	Easy Roast, Brambram, Couscous, Dessert
	Hard	Hard to roast or boil	Hard to Roast, Soursop, Hard Nature

In St. Vincent and Jamaica, breadfruit cultivars were distinguished on the basis of the most suitable method of preparation, roasting or boiling. This did not mean that cultivars could not be prepared using both methods and/or other methods of preparation. However, both roasting and boiling were popular in those countries, with roasting being more favored. Cultivars that were more suitable for roasting tended to roast easily and had great flavor and eating-quality. Cultivars that generally did not roast easily or did not have great flavor when roasted were better for boiling. After classifying cultivars based on the most suitable method of preparation, respondents further classified cultivars based on ease of cooking—easy or hard. Cultivars that were easy to roast or boil took less time than those that were hard to cook. This method of classification also contributed to cultivar names such as ‘Easy Roast,’ ‘Hard Nature’ and ‘Hard to Roast.’

## Discussion

The results of this study indicated that breadfruit was widely cultivated in home gardens and farms alongside other crops. The planting of breadfruit in home gardens showed that breadfruit was a valuable food crop for individual households, especially in rural communities that accounted for most respondents. However, other factors, such as the availability of adequate space for trees to grow, also likely influenced the prevalence and number of breadfruit trees in home gardens [7]. The practice of planting breadfruit trees in home gardens can be linked to the planting of breadfruit on provision grounds, which were designated areas on estates where the enslaved populations were allowed to grow their own food during the period of slavery [25, 26]. Although enslaved Africans did not initially favor breadfruit, it was still widely considered important for animal feed especially during periods of food crises such as after hurricanes and other natural disasters [6].

Provision grounds that consisted of mixed agriculture systems with a diversity of crops are the precursors of subsistence agriculture found throughout the Caribbean today [25]. Breadfruit was either planted on these provision grounds or on marginal lands but never in the main production area because it was not viewed as an economic crop. However, breadfruit has been important for household food and nutrition security, which is supported in the present study where breadfruit is cultivated for food, sharing with relatives, friends, and neighbors. The sharing of agricultural produce with relatives, friends and neighbors are part of the Anglophone Caribbean culture and is an inbuilt social security system. The countries in this survey share a similar economic and agricultural history including that of breadfruit which is supported by the fact that similar productions systems are used. As a border crop, breadfruit is used as a windbreak and shade for other more economically important crops. For example, in Trinidad and Tobago, breadfruit was often planted as a shade crop for cocoa (*Theobroma cacao*), which was grown mainly to be exported [8]. The production of breadfruit as a main crop is still not widespread throughout the region. However, this does not diminish its value and importance for food and nutrition security, which was underscored by most respondents who considered breadfruit important for either home consumption, for sale or both. These results are consistent with reports of increasing consumer appreciation and demand for breadfruit, which could eventually result in greater demand and production [7, 8, 23]. Breadfruit has been recognized by the International Treaty on Plant Genetic Resources for Food and Agriculture, which has listed it as one of the 35 priority crops to be conserved for food and nutrition security [27]. By documenting breadfruit cultivars, preparation methods and cultivar suitability for different uses, the folk nomenclature and traditional knowledge reported in this study can help to conserve breadfruit diversity in the Anglophone Caribbean. Preserving and transmitting traditional knowledge of the value and use of breadfruit for food and nutrition to the present and future generations and can encourage demand for consumption and increase its production and utilization in the region.

This study confirmed the depth of traditional knowledge of breadfruit biodiversity and traditional methods used to distinguish breadfruit types in the Anglophone Caribbean, which are important for conservation. Respondents used diverse traits related to agro-morphology, cooking methods, eating-quality, postharvest handling, and agronomic traits to identify, classify and describe breadfruit types. Although many vernacular names were recorded in different locations, the descriptions given suggest that the same or similar types were sometimes called by different names in different locations. Therefore, the range of breadfruit accessions and understanding the traditional system of classification are important because farmers and home gardeners over time develop skills to manage and select cultivars that they recognize. This could determine the range of diverse types they manage and conserve, which could eventually influence the evolution and adaptability of the crop [28].

The current study also showed that many factors could influence cultivar names. Some cultivars were named after people, places, other crop plants, ease of cooking, frequency of occurrence, food items and words used in a local dialect. Vernacular names such as ‘St. Kitts’ ‘Timor,’ and ‘England’ were all named after places. It was not clear when or the reasons the name ‘St. Kitts’ became a cultivar name, but it is likely based on informal distribution of planting materials among islands in the Anglophone Caribbean. The cultivar name ‘England’ showed a direct link to the role of the colonial government in the collection and introduction of breadfruit to the Anglophone Caribbean. Some misconceptions were found, which may be due to a lack of proper documentation of traditional knowledge. For example, in Jamaica, the name ‘Timor’ is believed to indicate the country where this cultivar was collected. However, descriptions provided by the curator of the St. Vincent Botanical Garden, who received breadfruit plants, indicated that the breadfruit from Timor had leaves with deep sinuses [3, 29]. From this description, it appears that ‘Cassava’ or ‘Captain Bligh’ are the cultivars linked to the country of Timor. Therefore, the cultivar name ‘Timor’ with dentate leaves, does not seem to be associated with the country Timor as accepted in some parts of Jamaica. Therefore, this study corroborates previous studies showing that traditional vernacular names of breadfruit based on morphological traits and morphological comparisons among cultivars remain very important in understanding breadfruit biodiversity in any geographic region [30]. Based on the documented history of breadfruit introduction and distribution in the Anglophone Caribbean, it is not expected that the high number of vernacular names uncovered in this, and previous studies reflect the true number of cultivars found in the region. Nevertheless, an understanding of the folk taxonomy and use of vernacular names is important to support future studies. Further studies using more reliable techniques such as molecular markers are needed to clarify the diversity and help identify synonymy among cultivars in the region.

Breadfruit production is an important part of the livelihood of many communities across the Anglophone Caribbean. It is of nutritional, socio-cultural, environmental, and economic importance and has a role in food and nutrition security in the region. This study explored the ethnobotanical and traditional knowledge associated with breadfruit in the Anglophone Caribbean islands and recorded the patterns or systems associated with identifying and distinguishing breadfruit types or cultivars and the understanding of the biodiversity that exists. Data collected from the respondents confirmed that there is an abundance of traditional knowledge associated with breadfruit biodiversity in the region.

## Conclusions and recommendations

Breadfruit vernacular names and systems of naming, describing, and classifying breadfruit types varied in different countries and within countries. Furthermore, clear morphological variations were observed, and in some cases, breadfruit types could be easily distinguished. However, some cultivars with the same names appeared morphologically different. Therefore, further studies are needed to understand the extent to which vernacular names represent genotypes that show distinct morphological, biochemical, and molecular characteristics in the Anglophone Caribbean. This could lead to a new and comprehensive classification scheme for breadfruit in the region and is important for conservation of the existing breadfruit germplasm.

## Data Availability

All data collected for this study were analyzed, interpreted, and included in this manuscript, but other datasets used and/or analyzed during the current study are available from the corresponding author on reasonable request.
